# Gender Classification Based on Geometry Features of Palm Image

**DOI:** 10.1155/2014/734564

**Published:** 2014-04-29

**Authors:** Ming Wu, Yubo Yuan

**Affiliations:** School of Information Science and Engineering, East China University of Science and Technology, Shanghai 200237, China

## Abstract

This paper presents a novel gender classification method based on geometry features of palm image which is simple, fast, and easy to handle. This gender classification method based on geometry features comprises two main attributes. The first one is feature extraction by image processing. The other one is classification system with polynomial smooth support vector machine (PSSVM). A total of 180 palm images were collected from 30 persons to verify the validity of the proposed gender classification approach and the results are satisfactory with classification rate over 85%. Experimental results demonstrate that our proposed approach is feasible and effective in gender recognition.

## 1. Introduction

Can you identify someone's gender through palm image? If you cannot, may be computer can help you. As an interesting yet challenging problem, classification of the gender of palm images is a rather novel research topic in computer vision. Automatic gender classification could be of important value in human-computer interaction, such as personal identification. Also, it is a useful preprocessing step for palm recognition. A computer system with the capability of gender classification has a wide range of applications in basic and applied research areas, including man-machine communication, security, law enforcement, demographics studies, psychiatry, education, and telecommunication.

Hand geometry, as the name suggests, refers to the geometric structure of the hand. Hand geometry-based systems are not new and have been available since the early 1970s. However, there is not much open literature addressing the research issues underlying hand geometry-based identity authentication; much of the literature is in the form of patents [1–3] or application-oriented description. Intensive academic research of this subject began in the late 90s. Golfarelli et al. [4] addressed the problem of performance evaluation in biometric verification systems. In that research they used prototypes based on hand shape and human face to prove their theory. In 2000 [5], another important paper was published. That was the first paper that addressed the identification problem based on hand geometry features with very satisfying results (97% identification accuracy). Formal recognition of this biometric characteristic came in 2004 [6]. Authors evaluated hand geometry as widely acceptable and easy collectable biometric characteristic.

Gender classification has been investigated from both psychological and computational perspectives. Although gender classification has attracted much attention in psychological literature [7–10], relatively few learning-based vision methods have been proposed, and most of this is based on face vision. A two-layer SEXNET is developed with 30∗30 pixel face (Golomb et al., 1991 [11]). The support vector machine is used to classify gender with low-resolution 21∗12 thumbnail faces (Moghaddam and Yang, 2002 [12]). An automatic real-time gender classification system is introduced that was based on LUT-Adaboost method (Wu et al., 2003 [13]). A novel method proposed for classifying the gender used local binary pattern (LBP) for face feature extraction (Sun et al., 2006 [14]). As the Gabor filters can extract the face features with different orientations and scales, it has strong representation ability. The mean Adaboost and local binary pattern methods are used for extracting the facial features (Makinen and Raisamo, 2008 [15]). A hybrid approach combining PCA and SFS (sequential forward selection) for face feature extraction is given by (Basmaci, 2011 [16]).

**Figure 1:**
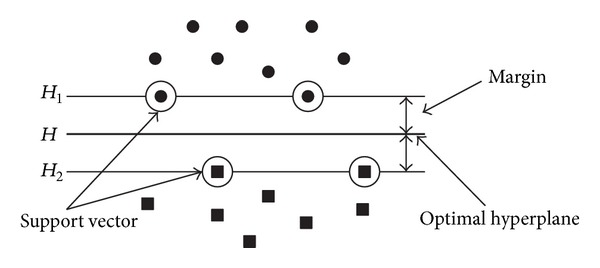
Support vector machine.

We use SVMs for gender classification of palm image in this paper. Support vector machine (SVM) is a universal classification algorithm proposed by Vapnik [17], which is regarded as a new innovation of learning machine based on the statistical learning theory. The basic theory of SVM can be depicted by a typical two-dimensional case shown in Figure 1, in which • and ■ denote two categories of samples, *H* is the separating hyperplane, and *H*_1_ and *H*_2_ are parallel to *H* and no training points fall between them.

The rest of the paper is organized as follows. In Section 2, database of palm images is described. In Sections 3 and 4, the methodology used for this study is described. In Section 5, the experimental results and the gender recognition method are discussed. And finally, the conclusion and future work are given in Sections 6 and 7.

## 2. Database of Palm Images

We performed the experiments on palm database set up by School of Information Science and Engineering, East China University of Science and Technology.

### 2.1. Acquisition Equipment

**Figure 2:**
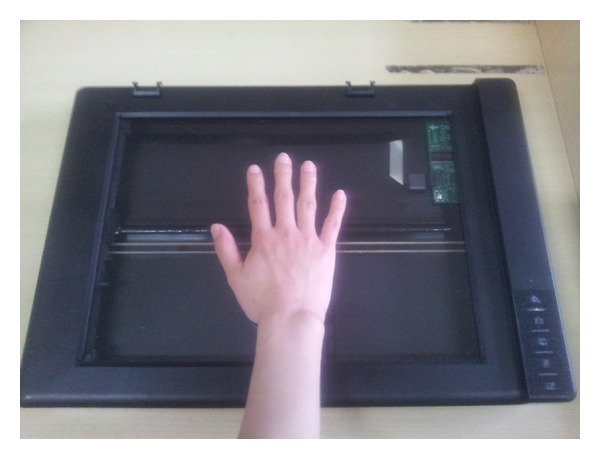
The palmprint data collection system.

A flatbed scanner, as a popular PC peripheral device, was used to acquire palm images. It possesses the benefits of high availability, uniform, and consistent good image quality, convenience, and low cost. In this work, the scanner that we used to acquire palm images was a BenQ (http://style.benq.com.cn/diy/k816/) as shown in Figure 2, which is mainly based on CIS (contact image sensor).

### 2.2. Database Description

This database contains palm images from 30 individuals. Since the two palms (right hand and left hand) of each person are different, we captured both and treated them as palm from different people. Each individual was asked to provide 3 left-hand palm images and 3 right-hand palm images, respectively. Thus, each person provides 6 images and the palm database contains a total of 180 images from 60 different palms. The size of all the original palm images is 2528∗1800 pixels with the resolution of 300 dpi. The individuals mainly consisted of student and staff volunteers from East China University of Science and Technology. Of the individuals in this database, 15 are male, and all the individuals are aged between 22 and 25.

**Figure 3:**
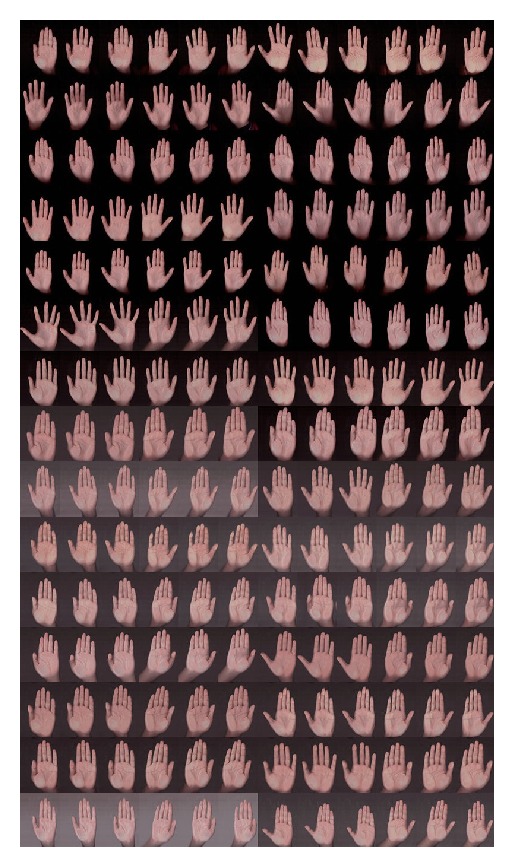
Database in thumbnails.

**Figure 4:**
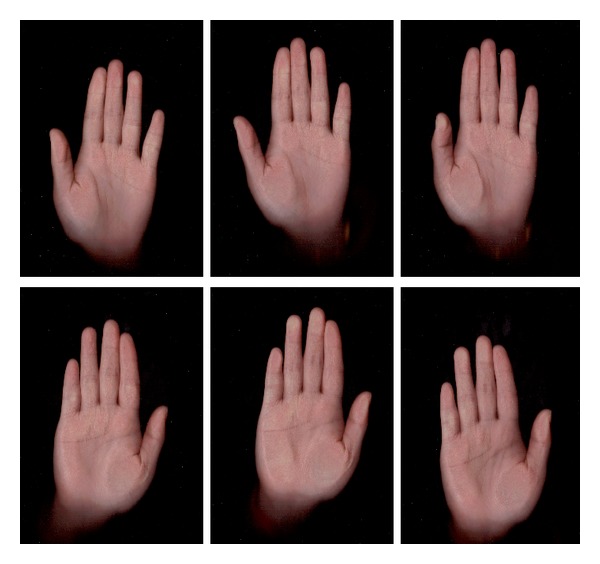
Palmprint images of both hands from female.

**Figure 5:**
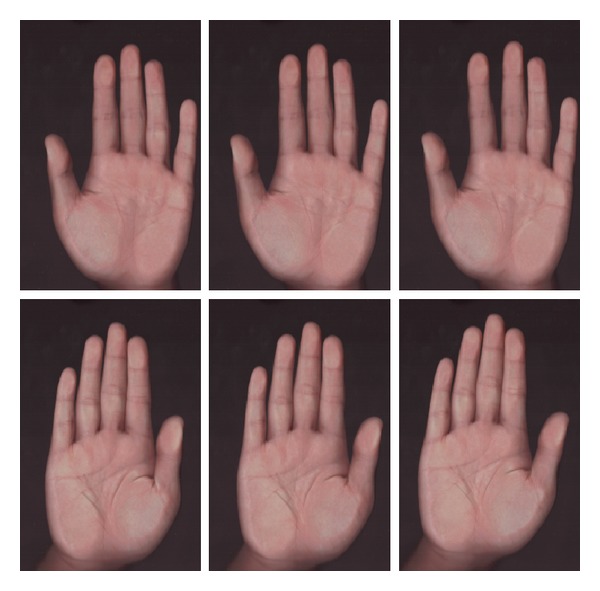
Palmprint images of both hands from male.

Figure 3 shows our database in thumbnails. Figures 4 and 5 show 3 left-hand palm images and 3 right-hand palm images from one individual.

After that, we got the final database as follows:

(1) $$\begin{array}{c} D=\left\{\left(I_{1}\left(x,y\right),l_{1}\right),\left(I_{2}\left(x,y\right),l_{2}\right),\ldots ,\left(I_{180}\left(x,y\right),l_{180}\right)\right\}, \end{array}$$

in which **I**_*i*_(*x*, *y*) denotes the *i*th palm image and (*x*, *y*)∈[*a*, *b*]×[*c*, *d*] is the geometry coordinate, *l*_*i*_ denotes the *i*th personal identification, in gender classification system, and *l*_*i*_ ∈ {1, −1} indicates male or female.

## 3. Preprocessing of Palm Images

**Figure 6:**
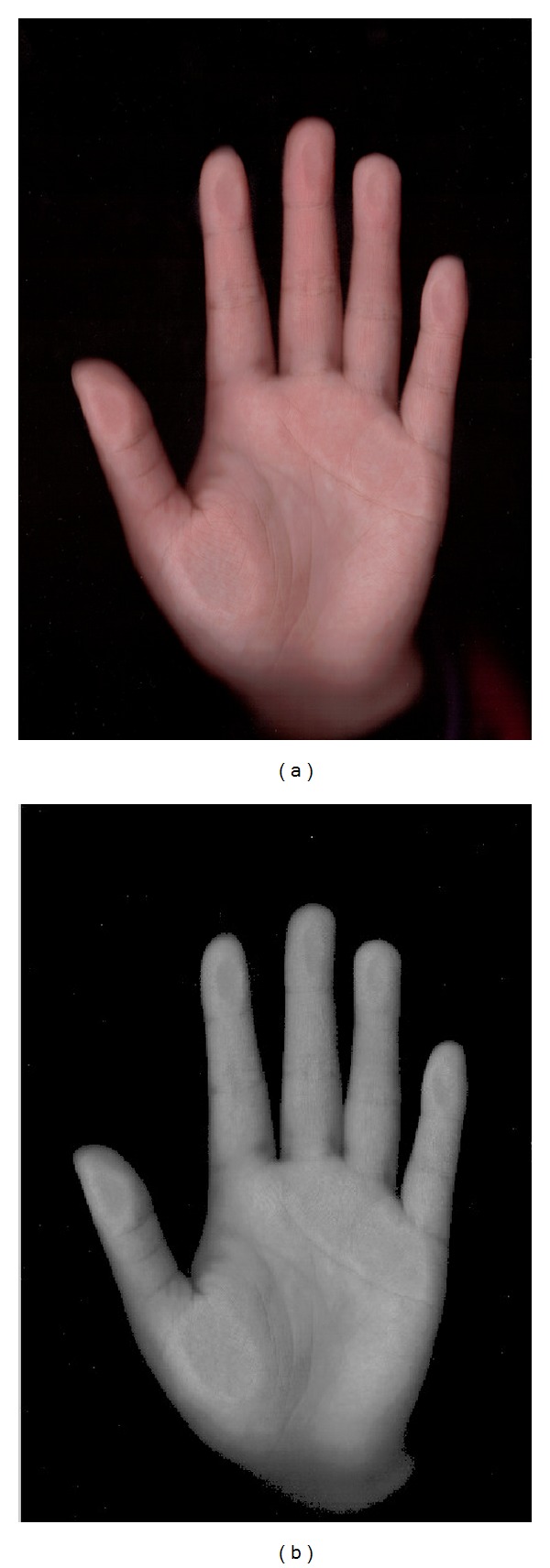
(a) The original image and (b) the gray image of (a).

As the illumination and the noise will make the feature extracting success rate drop quickly, so, original palm images cannot be used to extract hand shape features directly (Figure 6). In this paper, we propose a filter algorithm by analyzing palm image brightness features.

At the first step, we employ *m*-function (rgb2gray) and transfer the color palm image to gray image as follows:

(2) $$\begin{array}{c} g_{i}\left(x,y\right)=\text{rgb}2\text{gray}\left(I_{i}\left(x,y\right)\right). \end{array}$$

An illustration figure is shown as follows to indicate the basic changes after rgb2gray.

**Figure 7:**
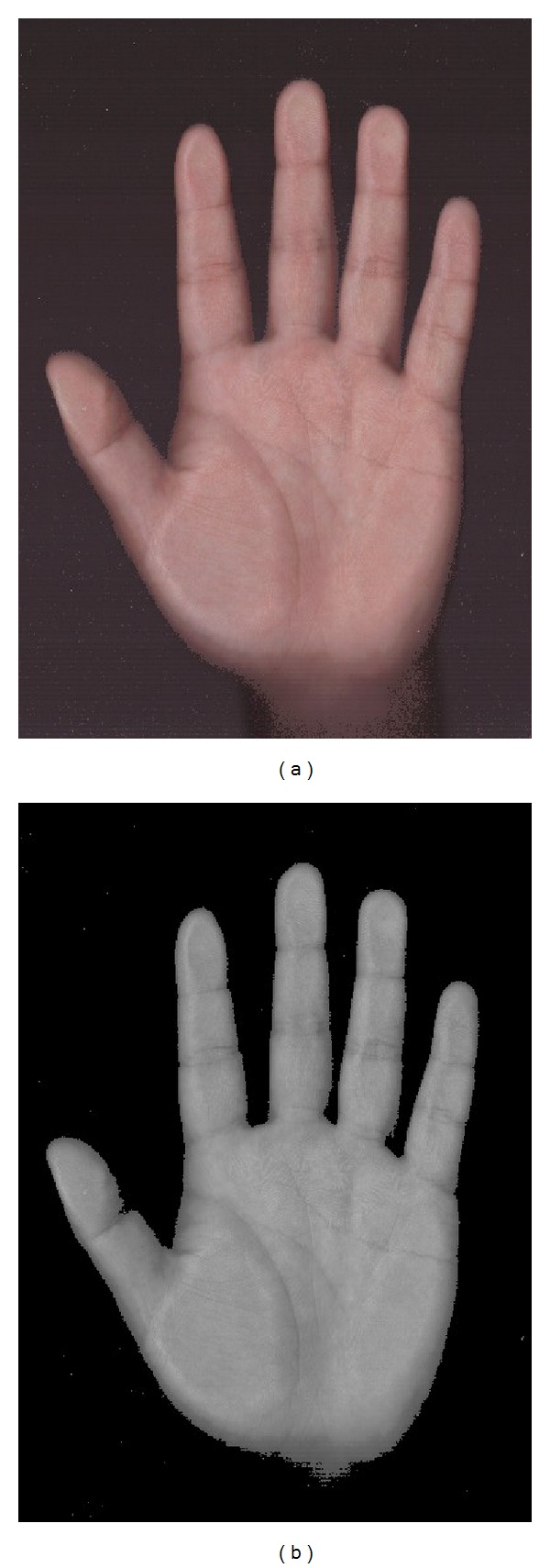
(a) The original image and (b) the filter image of (a).

The brightness feature is defined as the average of the intensity over the image:

(3) $$\begin{array}{c} H_{i}=\frac{1}{M*N}\overset{M}{\underset{x=1}{\sum }}\overset{N}{\underset{y=1}{\sum }}g_{i}\left(x,y\right), \end{array}$$

where *g*_*i*_(*x*, *y*) represents the gray value at pixel (*x*, *y*) of the *i*th palm image and *M* and *N* represent the numbers of rows and columns in the *i*th palm image. Then the response is filtered after threshold:

(4) $$\begin{array}{c} v_{i}\left(x,y\right)=\{\begin{array}{cc} g_{i}\left(x,y\right), & \text{if}\;g_{i}\left(x,y\right)\geq H_{i},\\ 0, & \text{otherwise}, \end{array} \end{array}$$

where *v*_*i*_(*x*, *y*) represents the new value at pixel (*x*, *y*).  Figure 7 shows the process of preprocessing an original palm image.

## 4. Geometry Features of Palm Images

The gender classification system relies on geometric invariants of a palm images. Typical features include length and width of palm images and aspect ratio of the palm.

As no pegs are used when capturing a palm image, the position, direction, and stretching degree may vary from time to time. In order to offset the effects of rotation and shift, Algorithm 1 was devised to compute the various feature values.

**Figure 8:**
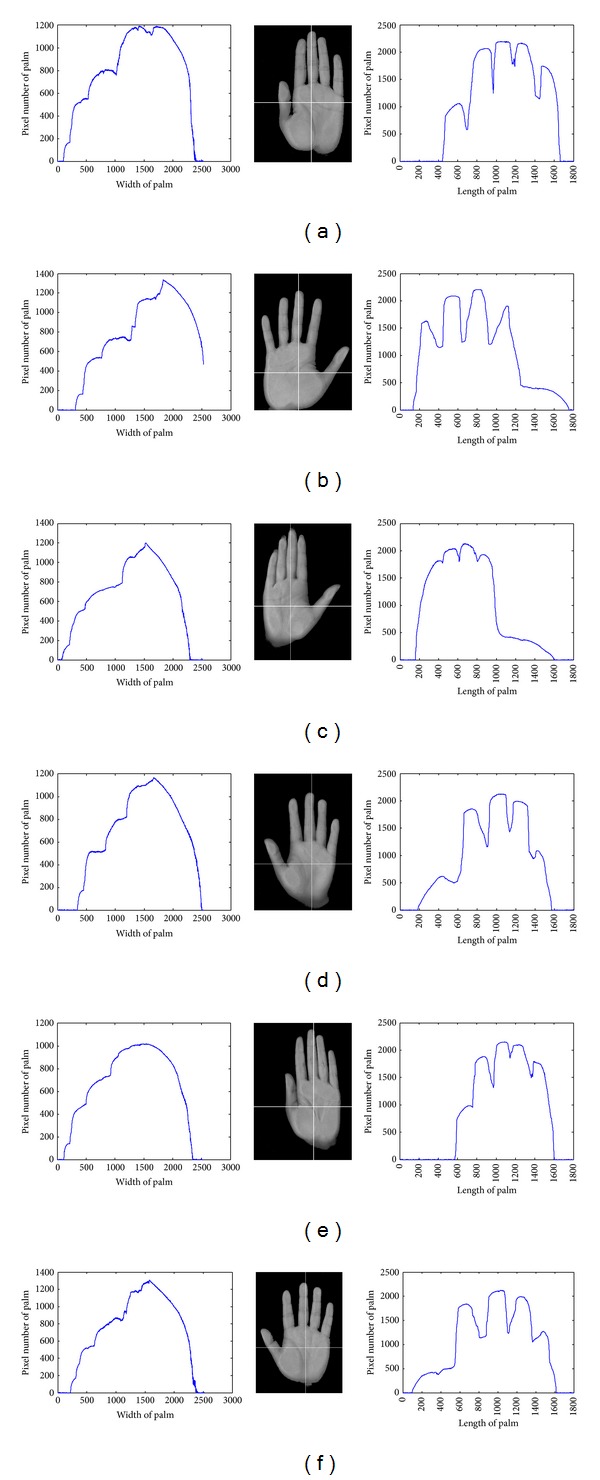
The length and width of palm.

Figure 8 shows the length and width of palm in which the geometry features mentioned above have been measured.

## 5. Classification Systems of Gender

The classification systems of gender was trained and tested using our own established database. Typical features include length and aspect ratio of the palm. First of all, feature values extracted by the above-described procedure are stored in a database after assigning gender. Then twenty percent of the database is randomly collected as the training samples and the rest as the testing samples. So, the numbers of training and testing samples are 36 and 114. Finally, polynomial smooth support vector machine (PSSVM) [18–21] is employed for training and classification.

Polynomial smooth support vector machine (PSSVM) is represented with the following optimization model:

(5) $$\begin{array}{c} \left(\omega^{*},\gamma^{*}\right)=\arg\underset{(\omega ,\gamma )\in R^{n+1}}{\min}F\left(\omega ,\gamma ,k\right), \end{array}$$

in which

(6) F(ω,γ,k)=ν2||h(e−(D(Aω−eγ)),k)||22+12(||ω||22+γ2),

in which

(7) $$\begin{array}{c} h\left(x,k\right)=\{\begin{array}{cc} x, & \text{if}\;x>\frac{1}{k},\\ -\frac{k^{3}}{16}{\left(x+\frac{1}{k}\right)}^{3}\left(x-\frac{3}{k}\right), & \text{if}\;-\frac{1}{k}\leq x\leq \frac{1}{k},\\ 0, & \text{if}\;x<-\frac{1}{k}. \end{array} \end{array}$$

The objective function of it is a quadratic differential and convex.

The method to solve the learning problem (5) can be seen in [18, 21].

**Figure 9:**
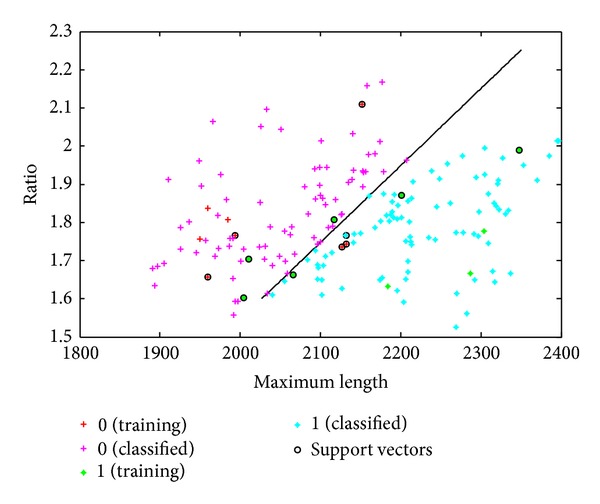
10% as train and 84.57% identification accuracy.

**Figure 10:**
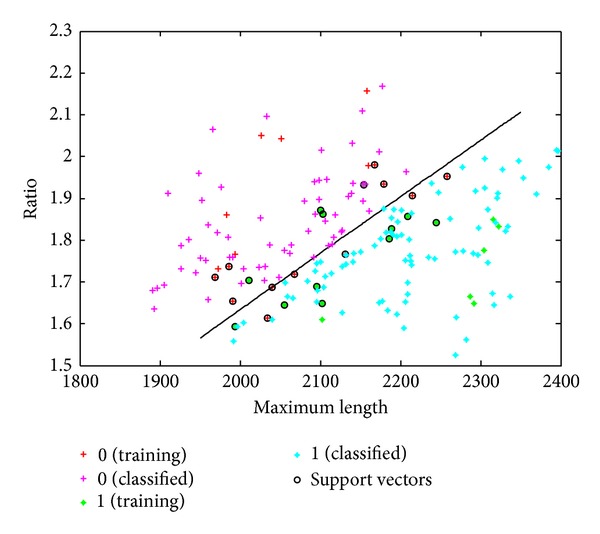
20% as train and 85.42% identification accuracy.

As you can see in Figures 9 and 10, the result is displayed intuitively, where 0 represents female and 1 represents male.

## 6. Conclusions

In this paper, a novel palm geometry-based biometric technique for gender classification using SVM has been proposed. It has been demonstrated that this biometric type uses simple technique and works quite well for gender recognition. Unlike other biometric approaches the proposed one does not use complicated methods, techniques, or procedure to attain high accuracy. Rather it uses fewer features than others and users can place their hands freely without needing pegs to fix the placement of their hand. Thus it would be convenient for practical implementation.

## 7. Future Works

Our ongoing work is investigating imaging setup, feature extraction, and a theoretical framework for matching. In particular, we are concentrating on the following problems: (i) the present imaging involves visible light. The charged coupled device (CCD) sensor has far better depth of field than the contact image sensor (CIS); it would be interesting to explore the effects of CCD scanner on the system performance; (ii) the existing feature set should be extended to include length and width of the fingers, aspect ratio of the fingers, and so forth; (iii) get more palm images of human being from different ages.

## Figures and Tables

**Algorithm 1:**
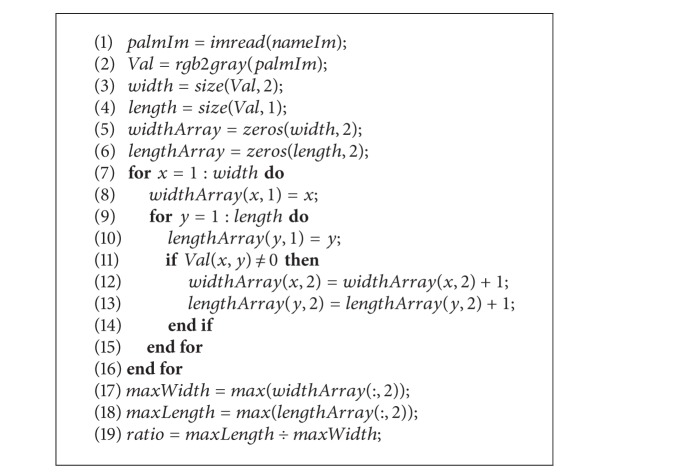
Extraction algorithm.

## References

[1] Ernst RH (1971). Hand ID system. *US Patent*.

[2] Jacoby OH, Giordano AJ, Fioretti WH (1971). Personal identification apparatus. *US Patent*.

[3] Lay HC (1971). Hand shape recognition. *US Patent*.

[4] Golfarelli M, Maio D, Maltoni D (1997). On the error-reject trade-off in biometrie verification systems. *IEEE Transactions on Pattern Analysis and Machine Intelligence*.

[5] Sanchez-Reillo R, Sanchez-Avila C, Gonzalez-Marcos A, Sanchez-Reillo R, Gonzalez-Marcos A (2000). Biometric identification through hand geometry measurements. *IEEE Transactions on Pattern Analysis and Machine Intelligence*.

[6] Jain AK, Ross A, Prabhakar S (2004). An introduction to biometric recognition. *IEEE Transactions on Circuits and Systems for Video Technology*.

[7] Bruce V, Burton AM, Hanna E (1993). Sex discrimination: how do we tell the difference between male and female faces?. *Perception*.

[8] Burton AM, Bruce V, Dench N (1993). What’s the difference between men and women? Evidence from facial measurement. *Perception*.

[9] Edelman B, Valentin D, Abdi H (1998). Sex classification of face areas: how well can a linear neural network predict human performance?. *Journal of Biological Systems*.

[10] O’Toole AJ, Vetter T, Troje NF, Bülthoff HH (1997). Sex classification is better with three-dimensional head structure than with image intensity information. *Perception*.

[11] Golomb BA, Lawrence DT, Sejnowski TJ (1991). SEXNET: a neural network identifies sex from human faces. *Advances in Neural Information Processing Systems*.

[12] Moghaddam B, Yang M-H Gender classification with support vector machines.

[13] Wu B, Ai H, Huang C Real time gender classification.

[14] Sun N, Zheng W, Sun C, Zou C, Zhao L, Wang J, Yi Z, Zurada JM, Lu B-L, Yin H (2006). Gender classification based on boosting local binary pattern. *Advances in Neural Networks—ISNN 2006*.

[15] Makinen E, Raisamo R (2008). Evaluation of gender classification methods with automatically detected and aligned faces. *IEEE Transactions on Pattern Analysis and Machine Intelligence*.

[16] Basmaci ES, Kaymakcioğlu U, Kurt Z Comparison of feature extraction and feature selection approaches to decide whether a face image belongs to a male or a female.

[17] Vapnik VN (1995). *The Nature of Statistical Learning Theory*.

[18] Yuan Y, Huang T (2005). A polynomial smooth support vector machine for classification. *Advanced Data Mining and Applications*.

[19] Liang J, Wu D (2005). A new smooth support vector machine. *Artificial Intelligence and Computational Intelligence*.

[20] Yuan YB (2012). Canonical duality solution for alternating support vector machine. *Journal of Industrial and Management Optimization*.

[21] Yuan Y, Fan W, Pu D (2007). Spline function smooth support vector machine for classification. *Journal of Industrial and Management Optimization*.

